# Who posted #MeToo, why, and what happened: A mixed methods examination

**DOI:** 10.3389/fpubh.2023.1060163

**Published:** 2023-03-06

**Authors:** Rose L. Siuta, Robert C. Martin, Kelly K. Dray, S.-N. Cindy Liu, Mindy E. Bergman

**Affiliations:** Department of Psychological and Brain Sciences, Texas A&M University, College Station, TX, United States

**Keywords:** sexual harassment, disclosure, mixed-method, #MeToo, reporting, gender

## Abstract

**Objectives:**

The #MeToo social media campaign raised awareness about sexual harassment. The purpose of the current study was to address three unexplored research questions. First, what factors influenced whether a person posted #MeToo? Second, how did posting (or not) influence participants' wellbeing? Finally, what motivated participants' posting (or not) #MeToo?

**Method:**

This mixed-methods study explores how #MeToo was experienced by full-time employees (*N* = 395) who could have posted #MeToo (i.e., experienced a sexual harassment event), whether or not they did so. Participants completed surveys in July of 2018 assessing social media use, sexual harassment history, relational variables such as relative power and social support, and job and life satisfaction. Participants also responded to open-ended survey questions about the context of and decisions about #MeToo posting.

**Results:**

Quantitative results indicated that sexual harassment history was the most powerful predictor of #MeToo posting, while power and interpersonal contact also contributed. Qualitative analyses (*N* = 74) using a grounded theory approach indicated themes associated with decisions to disclose, including feeling a responsibility to post, need for support, and affective benefits. Decisions not to disclose were event-related negative affect, posting-related negative affect, timing of the event, fit with the #MeToo movement, privacy concerns, and fear of consequences.

**Conclusion:**

This study contributes to the literature on sexual harassment disclosure by focusing on informal means of disclosure and drawing on comparisons to formal reporting and implications for workplaces. Online sexual harassment disclosure, in many ways, reflects the impediments to formal reporting procedures. Given the increased use of social media for purposes of disclosure, these findings suggests that organizations should recognize the legitimacy of sexual harassment reports made online and consider the possible failings of their formal reporting systems as reasons for online disclosure.

## 1. Introduction

The social media #MeToo movement went viral in Fall 2017 in the wake of sexual harassment and assault (SH) allegations against Harvey Weinstein. The MeToo movement, originated by Tarana Burke in 2006 and promoted by Alyssa Milano in 2017, urged sexual assault and harassment survivors to write “‘Me too' as a status [on their social media accounts]” in order to “give people a sense of the magnitude of the problem” (1, 2). Within the 1st day alone, over 500,000 people tweeted #MeToo, disclosing their own sex-based mistreatment and abuse (3). While most media coverage of the hashtag reported on celebrities (2, 4), Time's 2017 Person of the Year included women “span[ning] all races, all income classes, all occupations and virtually all corners of the globe” (5), who were named The Silence Breakers. However, criticisms have been raised about the motives, credibility, and ethics of sharing sexual harassment experiences on social media (6).

Previous research on sexual harassment disclosure has focused on the failings of formal reporting systems (7–10), while less research has explored the alternative forms that reporting may take, such as disclosures made online (11, 12). The current research aims to address this gap in the literature by exploring those disclosures made in the specific online context that followed the #MeToo movement. Given the proliferation of social media use and rise in disclosures made online, it is necessary to study how these processes are similar to, different from, and informed by shortcomings of a formal reporting process. Further, in order to make practical recommendations for managers it is important to understand the phenomenon of online sexual harassment disclosure in context and how these decisions align or diverge from decisions to formally report. In particular, given the increasing demand for organizations to respond to disclosures made through social media, it is crucial to first understand how and why individuals choose to make disclosures of workplace sexual harassment online, and what the potential impact of online disclosure may have on these individuals.

**Figure 1:**
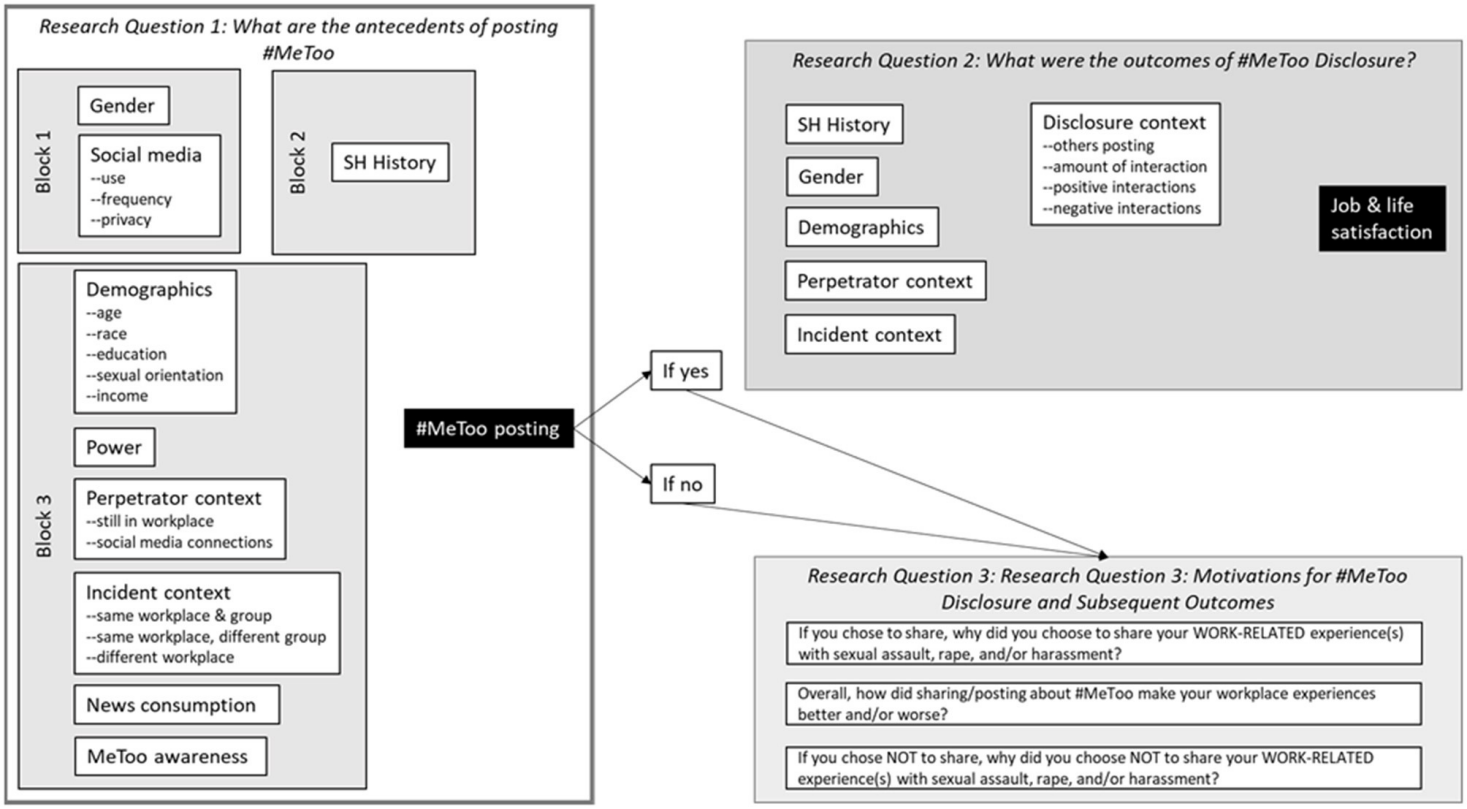
Research aims and factors explored for each research question.

The current study uses a mostly exploratory mixed-methods approach to (a) determine what individual, experiential, and social media-related factors precipitated #MeToo disclosures; (b) demonstrate how job and life satisfaction were impacted by #MeToo disclosures; and (c) uncover and describe the context and motivations for #MeToo disclosure and subsequent outcomes (see Figure 1). For our first aim, we explore incremental effects in predicting #MeToo disclosure from social media use, sexual harassment experiences, organizational power, current connections between the perpetrator and target, and demographic characteristics. For our second aim, we predict life and job satisfaction from the relevant variables described above as well as coworker reactions to the #MeToo post and the similarity between the current workplace context and when the SH event occurred. For our third aim, we use a grounded theory approach to analyze responses to open-ended questions regarding why participants chose to share using #MeToo and how sharing with #MeToo affected their workplace experiences. Throughout the paper, we draw on the limited literature about formal organizational reporting of SH, while making note of the differences between posting #MeToo and formal organizational reporting. At the outset, it is important to note that participants were required to have a sexual harassment experience that they could have posted about, but they did not have to post using #MeToo; thus, all members of our participant sample could have posted #MeToo, and we explored the factors as to whether they did (aim 1), and if they did, how that affected their life and job satisfaction (aim 2), and in their own words, what important motivations prompted their choice to disclose and what important outcomes occurred (aim 3).

### 1.1. Sexual harassment

Sexual harassment is defined as sex-based abuse occurring in the form of humiliating, derogatory, or coercive behaviors that can include sexual coercion, unwanted sexual attention, and gender harassment that ranges from demeaning comments to sexual assault and rape (13, 14). Cortina and Areguin (15), in their review of the sexual harassment in the workplace literature, emphasize that sexual harassment is more likely to be a put down (e.g., negative comments, obscene gestures, insults, and infantilization) than a come on (e.g., pressure for dates, unwanted touching on intimate body parts); at its core, sexual harassment is demeaning. Sexual harassment has negative effects on job and psychological wellbeing [(16); see Fitzgerald and Cortina (17) and Siuta and Bergman (18), for further reviews of the literature].

Sexual harassment experiences are not uncommon, with one meta-analytic estimate stating 58% of women have experienced workplace sexual harassment (19). In the United States 41% of women and 32% of men have experienced workplace sexual harassment (20). Lifetime prevalence rates of sexual harassment are similar in other parts of the world as well, with 33% of women in Australia and 45–55% of women in Europe experiencing sexual harassment at least once (21, 22). Higher prevalence of sexual harassment between 71 and 81% have been found in countries such as Denmark, Finland, France, the Netherlands, and Sweden, while lower rates between 24 and 32% have been found in Bulgaria, Poland, Portugal, and Romania (22). Even lower incidence rates have been found in Eastern nations like China and Japan at 12.5 and 9.5%, respectively (23, 24). Given the prevalence of sexual harassment across many countries, it is no surprise that becoming the target of sexual harassment is a common experience worldwide.

In this study, we use the term “sexual harassment” to encompass the behaviors of interest. The #MeToo hashtag and resurgence fundamentally concerns men in powerful positions within workplaces and the women who were abused by them (although, notably, it did not have to concern only male perpetrators and female targets), so sexual harassment is an appropriate term to represent this experience. Further, previous research found that workplace sexual assaults were frequently referenced in #MeToo posts (25), and sexual assault is a part of sexual harassment, so again the term sexual harassment seemed most appropriate.

### 1.2. Online disclosure before and during #MeToo

The #MeToo movement represents a shift in the ways that sexual harassment and violence are disclosed. Researchers have theorized that targets of sexual harassment and assault have turned to social media disclosure after being historically silenced by other reporting systems that fail to provide validation or support (25). Nevertheless, disclosure of sexual harassment and violence in an online setting was occurring prior to the #MeToo movement. One study addressing this type of disclosure prior to the #MeToo movement found that those posting online did so because they felt they had nowhere else to turn and that they received mostly supportive responses to their post (26). Additional studies confirm that social support, advice seeking, and storytelling are main outcomes and motivations behind social media disclosure of sexual abuse (27). Further, the digital space may provide a safer and easier space to engage in sexual harassment disclosures than in other offline contexts where tensions between everyday relationships, coworkers, and family may be more prevalent or distressing (28).

Research on the #MeToo movement has tended to focus on examining message content, including the specific incidents disclosed in posts (11, 12) and prevalence of support found in posts (29). However, there is little research examining the outcome of the act of posting with the hashtag, in particular in instances where workplace sexual harassment was disclosed. There is also little research exploring how power dynamics in the workplace played a role in individuals' decisions to disclose, or whether one's social media engagement is related to disclosure likelihood.

### 1.3. Why an exploratory study?

Although there are multiple literatures to draw upon herein, we think of this study as exploratory, rather than hypotheses testing or a mix of the two, for several reasons. First, although there were some relationships that we were confident would be robust and we could identify the direction of the relationship *a priori* (e.g., the effects of SH history and gender on #MeToo posting), we were not confident that these relationships would be predominant. Further, we were aware that some backlash (30) against the social media #MeToo campaign argued that the movement was led by a bunch of whiners [i.e., a new form of the long-standing and long-debunked whiner hypothesis, see Magley et al. (31)] and that it went too far in treating all men as predators (32), so we believed that allowing the data to speak for themselves without explicit hypotheses would best address these claims. Further, some relationships could plausibly be positive or negative because participating in #MeToo is risky (e.g., economically) but potentially rewarding (e.g., psychologically) in one way, but not participating was risky (e.g., psychologically) but potentially rewarding (e.g., economically) in another. Our review of the literature did not reveal which risks and rewards mattered to people. Additionally, while there is growing literature on the reporting of sexual harassment, some of this literature is methodologically suspect relative the *real life experiences* of the people who are harassed. For example, Hart (10) conducted an experiment to determine what stereotypes explained the negative reactions toward women who formally reported their sexual harassment experiences (or a coworker report on their behalf), but these experiments were conducted with “paper people” rather than real world experiences of persons embedded in on-going relationships with greater contextual knowledge of the harassment target. Further, it is clear that there is a complex calculus for deciding to formally report SH (9), and we anticipated similar complexity for #MeToo. Thus, we deployed a mixed methods study to explore the variables that have established effects on sexual harassment to determine how they played a role in social media disclosure and to create opportunities for people to describe, in their own words, the phenomenon of #MeToo relative to their own experiences.

### 1.4. Research question 1: Antecedents of #MeToo disclosure

Our first aim is to examine individual factors that influenced disclosure of SH using the MeToo hashtag. We explored several sets of predictors. As a first step, we investigated two factors, social media^1^ use and gender, that we expected to be inherently related to posting #MeToo on social media. We anticipated that people who used social media more would be more likely to post #MeToo for the simple reason of opportunity. Additionally, the #MeToo campaign was marketed as a women's movement from its beginning, which we believe may have led men to be less likely to use the hashtag. Further, sexual harassment has been linked to sex [i.e., women are more likely to experience sexual harassment than men; (17, 19, 33–36)], leading us to believe that gender would play an inherent role in decisions to post using #MeToo.

Next, we expected that decisions to post #MeToo reflected the totality of SH experiences. Despite the encouragement for people to post if they had any experiences of SH, we expected that people with more experiences would be more likely to post #MeToo. Further, we expected that SH experiences would provide incremental validity to our model, demonstrating that people's experiences with SH mattered in their disclosure above and beyond their social media use.

Third, we explored other factors that might add incremental validity to our model beyond SH experiences, gender, and social media use: (a) the relative organizational power of the SH perpetrator compared to the target, (b) whether the perpetrator and #MeToo poster were still connected, and (c) demographic characteristics.

Relative power refers to the power dynamics between those who harass and those who become the targets of harassment and can include perpetrators who have (a) greater, (b) equivalent, or (c) lower organizational power than the targets (37). Relative organizational power was considered because SH is typically enacted by more powerful people toward less powerful people (7, 38), but reporting more powerful people is more threatening to workplace and economic wellbeing than reporting less powerful people, and it is clear that there are economic costs to reporting (8). Further, within the context of formal reporting, high status perpetrators are less likely to receive organizational repercussions for their harassing behaviors (7). Despite this, some evidence suggests that reporting is positively associated with the perpetrator's power (7).

We also examined whether the perpetrator and #MeToo poster were still connected. Because #MeToo was a report of lifetime incidence, it is likely that some events that precipitated postings happened years if not decades before whereas others happened mere days or hours ago. Thus, there were likely to be differences in on-going connection between perpetrator and target (e.g., still coworkers and social media connections), which would likely influence #MeToo declarations due to the repercussions varying widely across the connection levels between the two. In our exploration, we were interested in describing how #MeToo disclosure decisions were influenced *via* social network connections maintained through their personal social media accounts. On one hand, individuals might be more likely to post using #MeToo if they knew that their perpetrator was not able to view and comment on their post because of the safety that would be afforded to the person without these connections. On the other hand, a theme in #MeToo disclosures in the media was the motivation of bringing justice to bear (whether in legal courts or the court of public opinion) onto those who engaged in sexual harassment; thus, those who shared with #MeToo might have been more likely to do so if they knew their perpetrator was able to see the post. Although we believed the former to be more likely than the latter, we could not predict the direction of this relationship with certainty.

Finally, we examined a host of demographic variables that have been linked to SH [for a review, see Cortina and Areguin (15)]. Sexual harassment has been linked to race [racially minoritized individuals–such as BIPOC–are more likely to experience sexual harassment than are White individuals; (39, 40)], sexual orientation [people identifying as LGBTQ are more likely to experience sexual harassment than heterosexual or cisgendered individuals; (41)], and age [younger people are more likely to experience SH than older people; (42)]. Further, people who have more than one minoritized identity (e.g., Black women) are more likely to experience sexual harassment than those who occupy only one minoritized identity (e.g., Black men or White women) or none [(43); see Minnotte and Legerski (44), for a review of intersectional identities and SH]. The goal of this part of the exploration was to examine whether these minoritized identities lead to more #MeToo reports (due to the need to express frustration over both the harassment itself and the higher rate of harassment experienced) or fewer (due to the need to protect against risks to workplace and economic wellbeing).

### 1.5. Research question 2: Outcomes of #MeToo disclosure

Our second aim is to determine how posting #MeToo was related to a person's job and life satisfaction, restricting our sample to only those people who posted #MeToo on social media. We reexamined several factors in the #MeToo experience described in our first research aim.

First, because all of the participants in this study experienced some SH (because they had to be able to post #MeToo per our recruitment protocol), it was important to include history of SH experience as an initial step because there is a robust link between SH and job and life satisfaction (14, 16, 45–52).

We also examined whether several contextual variables would predict job and life satisfaction beyond SH history. First, we examined whether the discloser and perpetrator were still connected. The continued connection could influence job and life satisfaction because it reminds targets of their SH incident, puts them at risk for continued SH, or indicates that the organization has allowed the perpetrator to remain (53). Additionally, we examined the extent to which the workplace is similar to what it was when the SH happened (i.e., same workplace and workgroup, same workplace but different workgroup, and different workplace). Like current connections with the SH perpetrator, this variable was aimed at assessing the extent to which the target was still embedded with systems and processes that were part of the SH experience.

Social support is a critical factor in stress management (54, 55) and was therefore expected to be related to job and life satisfaction in the context of #MeToo posts. Thus, we examined reactions from coworkers. This included both positive and negative reactions following #MeToo posts as well as the extent to which others shared their own #MeToo experiences; For instance, negative reactions to #MeToo posts could leave posters feeling unsupported by their coworkers while positive reactions to #MeToo posts indicate better social support. We also considered whether the extent to which one's social media connections also shared #MeToo experiences would influence job and life satisfaction and again considered competing relationships. A person who has many social media connections that shared #MeToo experiences may feel greater job and life satisfaction due to a greater sense of support from knowing others have faced a similar situation. On the other hand, a person who sees that many of their social media connections have experienced similar situations may be more likely to feel a sense of dissatisfaction with their workplace for allowing sexual harassment to spread to so many individuals.

Finally, we also examined demographic characteristics (e.g., gender, race, education, and income) as predictors of how #MeToo disclosure affected job and life satisfaction. We included these variables in our analysis to determine if they provided incremental validity in predicting job and life satisfaction above and beyond the other factors. There is some research indicating differences across demographic groups on job and life satisfaction, but many of these differences disappear when differences in stressors across groups are accounted for (56, 57).

### 1.6. Research question 3: Motivations for #MeToo disclosure and subsequent outcomes

Our third research aim was to understand what motivated individuals in their choices to disclose or not disclose using #MeToo and to uncover outcomes of these disclosure decisions other than job and life satisfaction. To address this research question, we examined qualitative responses to questions probing for why an individual chose to disclose or not to disclose using #MeToo, and how disclosure affected subsequent workplace experiences. The goal of our mixed-methods approach was to empower our participants to share the important outcomes of #MeToo disclosure in their own words, and to give a more detailed picture of the lived experiences of those making #MeToo disclosure decisions. A grounded theory approach was used to analyze, code, and arrange responses into categories (58, 59).

## 2. Method

An online survey was conducted *via* Qualtrics on Amazon Mechanical Turk. Approval of the research protocol was obtained from Texas A&M University's Human Subjects Protections Program prior to recruitment and data collection. Consistent with reporting standards, all measures, manipulations, sample size, and data exclusions are reported. All data, materials, and analysis code are available by emailing the corresponding author. Data were analyzed using IBM SPSS v25 (60) and R v4.0.3 (61). The analysis and design for this study were not pre-registered.

### 2.1. Statistical considerations

A sample size of ~400 was determined *a priori* to be necessary to capture participants in the two outcome groups (i.e., those who did disclose with #MeToo and those who did not disclose with #MeToo). Non-disclosure of sexual harassment is far more common than disclosure in formal reporting contexts (62, 63), with estimates of reporting rates below 10%. We chose a sample size of 400, such that even if only 10% of these participants disclosed using #MeToo there would still be an adequate sample size to capture variability for this disclosure group.

### 2.2. Data collection and cleaning

We collected data through Amazon Mechanical Turk in July 2018 (~9 months after Alyssa Milano urged sexual assault and harassment survivors to tweet #MeToo). Participants viewed a posting on the platform that described the study and then elected to enter the survey platform, where they viewed the consent form that described the study, the associated risks, and the associated benefits of participation. To qualify for inclusion in the study, all participants were required to be at least 18 years old, employed within the U.S., and working full-time (30+ h per week). In addition, to be included in the study, we specifically recruited employees who had knowledge of the #MeToo campaign and had an experience with sexual harassment or assault that they considered sharing on social media (i.e., anyone in our sample could have reported #MeToo, but were not required to have done so). Two questions determined inclusion based on #MeToo knowledge and opportunity to report using #MeToo (*Were you aware of the #MeToo campaign?* and *Did you consider sharing your experiences with sexual assault or harassment during the #MeToo campaign?*). Participants were paid $2.00 for completing the ~20 min survey.

Because the use of artificial intelligence on this crowd-sourcing platform has been an increasing concern for researchers [e.g., (64)], we excluded data from our analyses when there was evidence that it had been given by bots (computer programs used to automatically complete Mturk tasks) (65). Two trained undergraduate research assistants independently coded each survey response for the likelihood of it having been completed by a bot, with obvious bots being screened and rejected (65). The criteria for such a determination were: (a) participant copies the question being asked and pastes it into the textbox as their answer, (b) two or more participants submit identical responses to qualitative questions, (c) participant copies text from a website and pastes it as their answer, or (d) participant provides an answer that is inconsistent with the question asked. The coders agreed on 92.4% of the responses. When the coders disagreed, a third coder [an Industrial/Organizational (I/O) Psychology Ph.D. student] reviewed the response and made a final determination.

### 2.3. Participants

Of the 487 responses to our survey, our data cleaning procedures left us with a sample of 395 participants (81.11% of original sample). Participants had an average age of 31.95 (SD = 8.51; see Table 1) and were majority White (64.05%), followed by Asian/Asian American (15.19%), Black/African American (9.62%), Hispanic/Latinx (6.84%), American Indian/Alaska Native (3.04%), and individuals who preferred to self-describe (1.27%). The majority of participants were women (59.75%), followed by men (38.73%), and non-binary/third gender individuals (1.27%). Finally, participants were majority heterosexual (81.52%), followed by bisexual (9.87%), homosexual (4.31%), asexual (2.03%), and prefer to self-describe (0.76%). Of this sample, 74 participants (18.73%) reported using the MeToo hashtag on social media.

**Table 1:** Sample demographic characteristics.

	**Demographic characteristic**	***n***	**%**
Gender	Man	153	38.73
	Woman	236	59.75
	Non-binary/third-gender	5	1.27
	Prefer not to say	1	0.25
	Total gender	395	100.0
Race	White/Caucasian	253	64.05
	Black/African American	38	9.62
	Asian/Asian American	60	15.19
	Hispanic/Latino	27	6.84
	American Indian/Alaska Native	12	3.04
	Prefer to self-identify	5	1.27
	Total race	395	100.0
Sexual orientation	Gay	5	1.27
	Lesbian	12	3.04
	Bisexual	39	9.87
	Heterosexual	322	81.52
	Asexual	8	2.03
	Prefer to self-identify	3	0.76
	Prefer not to say	6	1.52
	Total sexual orientation	395	100.0

### 2.4. Procedure

After reporting their demographic information, participants reported on their experiences with sexual harassment. Next, participants were asked if they shared their experience(s) with sexual assault, rape, and/or harassment on a social media account with the #MeToo hashtag, and completed measures regarding their social media use, relative organizational power, and connections with the perpetrator(s). Participants who shared their experience(s) with sexual assault, rape, and/or harassment on a social media account with the MeToo hashtag were then asked to complete measures related to (a) job satisfaction, (b) life satisfaction, and (c) interpersonal interactions related to their social media post.

### 2.5. Measures

#### 2.5.1. Sexual harassment

Participants completed the 21-item version of the Sexual Experiences Questionnaire [SEQ; adapted to ask about sexual harassment experiences over their lifetime; (66, 67)]. Participants were instructed to indicate how frequently their co-workers and/or supervisors engaged in various inappropriate behaviors at work on a 5-point Likert scale (0 = never, 4 = many times). Some example items include “...implied faster promotions or better treatment if you were sexually cooperative?” and “...publicly addressed you in unprofessional terms (e.g., honey and babe)?” This scale showed acceptable reliability (α = 0.96).

#### 2.5.2. Social media use

Participants completed a 6-item adapted measure about social media use (68). Participants were asked to respond to the 6-item scale on a 7-point Likert scale (1 = strongly disagree, 7 = strongly agree). Example items include “I use social media to find and spread information,” “I use social media to keep abreast of current events,” and “Social media is primarily for socializing.” This scale demonstrated adequate reliability (α = 0.79).

#### 2.5.3. Social media use frequency

Participants completed an additional item regarding the frequency of their social media use, which asked “How often do you use social media?” Answer choices ranged from “hourly” to “never.”

#### 2.5.4. Social media privacy

Participants were asked “Is your preferred social media account private or public?” to assess if their social media postings were viewable to those outside of immediate connections. Response options were “public” (coded as 0) and “private” (coded as 1).

#### 2.5.5. Relative organizational power

To assess participants' relative organizational power compared to the perpetrators who sexually harassed, raped, or sexually assaulted them, they were asked “In relation to yourself, does the perpetrator of the sexual harassment, rape, and/or assault have more, less, or equal power in the organization?” Response options included “more power in the organization than you” (coded as 0), “less power in the organization than you” (coded as 1), and “equal power in the organization than you” (coded as 2).

#### 2.5.6. Same workplace as the perpetrator

Participants were asked “Are you still in the same workplace as the perpetrator?” in order to assess their current level of connection with the perpetrator. The response options were “yes” (coded as 0) and “no” (coded as 1).

#### 2.5.7. Location of SH

To assess the overlap between the participant's current workplace and the workplace in which the sexual harassment, rape, or sexual assault occurred, participants responded to the following question: “Are you still at the workplace where these events happened, or a new one?” Response options were “current workplace, same workgroup” (coded as 0), “current workplace, different workgroup or worksite” (coded as 1), and “previous workplace” (coded as 2).

#### 2.5.8. Interactions regarding post

Participants who indicated that they had used the MeToo hashtag were asked “After posting with the #MeToo hashtag, did people from your workplace reach out to discuss your post with you (e.g., *via* personal message, email, in-person, commenting on your post)?” Response options were “yes” (coded as 0) and “no” (coded as 1). If participants responded in the affirmative, they were then asked “How many people interacted with you negatively as a result of your #MeToo disclosure? If you don't know, please estimate” and “How many people offered positive support? If you don't know, please estimate.” Next, these participants were asked “After you shared your #MeToo post, did people share their stories with you?” Response options were “yes” (coded as 0) and “no” (coded as 1).

#### 2.5.9. Job satisfaction

Participants also completed an adapted 3-item job satisfaction measure (69) using a 7-point Likert scale (1 = strongly disagree, 7 = strongly agree). Items from the scale include “Since the #MeToo movement began, I have… been satisfied with my job,” “... not liked my job” (reverse-coded), and “... not liked working here” (reverse-coded). This scale demonstrated suboptimal reliability (α = 0.57), so the item most negatively impacting Cronbach's alpha was dropped from the scale, improving reliability to an acceptable level (α = 0.91).

#### 2.5.10. Life satisfaction

Participants also completed an adapted 5-item life satisfaction measure (70) using a 7-point Likert scale (1 = strongly disagree, 7 = strongly agree). Some example items from this scale include “Since the #MeToo movement began, I have been satisfied with my life” and “... felt that if I could live my life over, I would change almost nothing.” This scale demonstrated adequate reliability (α = 0.84).

#### 2.5.11. Qualitative questions

Qualitative questions were included in the survey to gain a greater depth of information about why participants chose to use #MeToo or not, and about the outcomes experienced from participating in the #MeToo social media campaign. Because online disclosure of sexual harassment is a relatively new phenomenon, we used these qualitative questions to further inform the reasons behind #MeToo participation. This allowed for an accumulation of organizational outcomes besides job and life satisfaction to emerge from participant accounts. Participants who indicated posting #MeToo were asked to respond to two open-ended questions: (1) “If you chose to share, why did you choose to share your WORK-RELATED experience(s) with sexual assault, rape, and/or harassment?” and (2) “Overall, how did sharing/posting about #MeToo make your workplace experiences better and/or worse?” Participants who indicated not posting #MeToo (even though they could have), were asked: “If you chose NOT to share, why did you choose NOT to share your WORK-RELATED experience(s) with sexual assault, rape, and/or harassment?”

### 2.6. Qualitative coding procedure

A grounded theory approach was used to analyze the data (58). Grounded theory attempts to uncover phenomenological processes to develop a theory of cause and consequence of the phenomenon of interest. In this case, there is already some knowledge regarding formal reporting of sexual harassment and assault in workplace contexts, but a new point of view is warranted given that the #MeToo movement involves informal reporting in non-workplace environments (i.e., on social media). First, a trained coder (a White female undergraduate research assistant in an I/O Psychology lab) read the responses to each question in their entirety to gain an understanding of the entire response provided by each participant as a whole. Next, the trained coder completed open, line-by-line coding with the aim of identifying key ideas and themes, focusing on the specific text as written and without implying broader structure (such as relationships between these themes) within the data (58). This initial open coding process ensures that the data analysis and theoretical development is guided by the words contained in the collected responses and not forced to adhere to a predetermined theoretical framework (58, 59). After this initial open coding process, the undergraduate research assistant and the first author of this paper (a White female I/O Psychology Ph.D. student) collapsed these codes into broader categories in order to reduce redundancy (e.g., “mad” and “angry” would be collapsed into a single code). Then we did axial coding, during which the coders considered how the categories from the open code process fit into the paradigmatic model of grounded theory, which a priori specifies a set of components for a model. Briefly, these subcomponents are: causal conditions (i.e., anything that predicates the occurrence of the development of a phenomenon); phenomenon (i.e., main events or incidents that subsequent actions are related to); context (i.e., properties related to the phenomenon); intervening conditions (i.e., factors that interact with or bear action on the phenomenon); actions/strategies (i.e., responses that occur in order to manage the experience of the phenomenon), and consequences (i.e., outcomes of the actions/interactions). The goal of axial coding is to demonstrate how each code is related to the central phenomenon of interest by determining which subcomponents of the paradigmatic model each code belongs to. For example the code *privacy concerns* may be related to the phenomenon of #MeToo disclosure decisions because it belongs to the subcomponent of intervening conditions, meaning that privacy concerns were one factor participants considered to contribute to their decisions to either disclose or not disclose using #MeToo. Each of the final codes was considered in relationship to the phenomenon of #MeToo disclosure and ultimately three axes were developed: context, contributing reasons for disclosure, and consequences.

## 3. Results

### 3.1. Model 1: Antecedents of #MeToo disclosure

Our first aim was to examine the factors related to #MeToo reporting on social media. We conducted hierarchical logistic regression to predict #MeToo reports (yes/no) by: gender and characteristics of social media use (Model 1.1); history of SH experiences (Model 1.2); and, perpetrator power, connections to the perpetrator, and demographics (Model 1.3; see Table 2). The final Model 1.3 included 373 participants (11 participants were excluded for incomplete data, and 11 participants were removed due to low sample size in questions of gender and sexual orientation) and was statistically significant [$\chi_{\left(20\right)}^{2}$ = 86.02, *p* < 0.001], with a Nagelkerke *R*^2^ of 0.34 and correctly classified 84.7% of cases (Table 3).

**Table 2:** Results from hierarchical logistic regression.

	**Model 1**				**Model 2**				**Model 3**			
**Variable**	**B**	**SE**	**Sig**.	**Odds ratio**	**B**	**SE**	**Sig**.	**Odds ratio**	**B**	**SE**	**Sig**.	**Odds ratio**
Constant	−0.97	0.85		0.38	−2.11	0.89	^*^	0.12	−0.35	1.28		0.70
Gender	0.19	0.29		1.21	0.33	0.31		1.39	0.69	0.35	^*^	2.00
*(base = Man)*												
Social media use	0.05	0.14		1.05	−0.01	0.14		0.99	0.08	0.17		1.08
Frequency of social media use	−0.16	0.14		0.85	−0.15	0.14		0.86	−0.27	0.16		0.76
Social media privacy	−0.97	0.28	^***^	0.38	−1.02	0.30	^**^	0.36	−0.69	0.34	^*^	0.50
*(base = Public)*												
SEQ					0.05	0.01	^***^	1.05	0.04	0.01	^***^	1.04
Age									−0.03	0.02		0.97
Race									0.15	0.33		1.17
*(base = White/Caucasian)*												
Sexual orientation									−0.33	0.45		0.72
*(base = Heterosexual)*												
Education									−0.31	0.46		0.74
*(base = College and more)*												
Income									−0.05	0.09		0.95
Incident context											^*^	
*Current workplace, different workgroup*									−0.93	0.44	^*^	0.40
*Previous workplace*									−1.41	0.52	^**^	0.25
*(base = Current workplace, same workgroup)*												
Current perpetrator context									0.68	0.46		1.98
*(base = In current workplace)*												
Organizational power												
*Perpetrator has less power*									0.29	0.42		1.34
*Perpetrator has equal power*									−0.98	0.45	^*^	0.38
*(base = Perpetrator has more power than target)*												
SM connection to perpetrator									−0.53	0.46		0.59
*(base = Yes)*												
SM connection to past perpetrators												
*No*									−0.61	0.48		0.54
*I have no other perpetrators*									−0.29	0.64		0.75
*(base = Yes)*												
MeToo awareness									−0.04	0.19		0.96
News consumption									0.05	0.26		1.05

N = 373.

* *p* < 0.05,

** *p* < 0.01,

*** *p* < 0.001.

**Table 3:** Results of omnibus tests of model coefficients.

**Model**	**Chi-square**	**df**	**Sig**.	**Total chi-square**	**Total df**	**Total Sig**.	**Total Nagelkerke R square**
1	14.61	4	^**^	14.61	4	^**^	0.06
2	43.26	1	^***^	57.87	5	^***^	0.23
3	28.15	15	^*^	86.02	20	^***^	0.34

* *p* < 0.05,

** *p* < 0.01,

*** *p* < 0.001.

Odds ratios revealed that women were two times more likely to post than men (*p* < 0.05).^2^ Measures of social media usage and frequency did not reach significance, however social media privacy was a significant predictor of #MeToo disclosure (*p* < 0.05). Those who reported having public social media page were two times more likely to post #MeToo than those with a private social media page.

SH experiences were added to the model in Block 2 and significantly predicted #MeToo disclosure (*p* < 0.001) above and beyond gender and social media factors [Block 1.2 $\chi_{\left(1\right)}^{2}$ = 43.26, *p* < 0.001; Model 1.2 $\chi_{\left(5\right)}^{2}$ = 57.87, *p* < 0.001]. For each additional increase in SEQ score, participants were 1.04 times more likely to post using #MeToo.

Block 3 then added factors related to perpetrator power, connections to the perpetrator, and demographics to the model. Working in the same place as the perpetrator (*p* < 0.05) and the relative organizational power of the perpetrator (*p* < 0.05) predicted #MeToo disclosure above and beyond the factors entered in previous blocks [Block 1.3 $\chi_{\left(15\right)}^{2}$ = 28.15, *p* < 0.05; Model 1.3 $\chi_{\left(20\right)}^{2}$ = 86.02, *p* < 0.001]. Odds ratios revealed that people who were currently employed in the same workplace and same workgroup where the SH occurred were 2.53 times more likely than those who were currently in different workgroups (but same workplace) as where the SH occurred, and 4.08 times more likely than those who were currently in different workplaces than where the SH occurred to post using #MeToo. Lastly, those who had a perpetrator with more organizational power than themselves were 2.65 times more likely to post using #MeToo than those who had a perpetrator with equal organizational power.

In sum, SH experiences and social media privacy predicted #MeToo posts. Perpetrator power and incident context incrementally predicted #MeToo, whereas demographics did not.

#### 3.1.1. Relative weights analysis

In order to determine the relative importance of the predictors contributing the most to our model, a relative weights analysis (71) was conducted for Research Question 1. Relative weights analysis is used to show the proportional contribution of individual predictor variables on the total variance accounted for in the regression model (72). Unlike standardized regression coefficients, relative weights estimates the importance of a variable by taking into account the unique contribution of a predictor variable, as well as its contribution when combined with other predictor variables (71). The analysis was completed using the R software package RWA Web [see Tonidandel et al. (72)]. Following Tonidandel et al.'s (73) recommendations, bootstrapping with 10,000 replications was used to produce 95% confidence intervals and significance tests. The analysis showed that SEQ scores accounted for 35.43% of the explained variance in #MeToo disclosures, which was the largest contributing variable. The social media connection to the perpetrator accounted for 10.60%, and the similarity of the incident context to one's current workplace context accounted for 10.94% of the explained variance in #MeToo disclosures. Lastly, social media privacy accounted for 8.79%, and perpetrator organizational power accounted for 9.35% of the explained variance in #MeToo disclosures. A further examination of the relative weights showed that only SEQ scores, social media connection to the perpetrator, and proximity of the incident context to one's current workplace context explained a statistically significant amount of variance in #MeToo disclosures. Because social media connection to the perpetrator emerged as a statistically significant predictor while gender, social media privacy, and relative organizational power of the perpetrator were not significant, the results of the relative weights analysis differ somewhat from the results of the logistic regression analysis. It is not unusual for relative weights significance to differ from regression coefficient significance because these statistics address different research questions (73).

### 3.2. Model 2: Outcomes of #MeToo disclosure

We conducted separate hierarchical regressions to predict job satisfaction, and life satisfaction. Both equations included several blocks of predictors: (1) SH experiences; (2) interpersonal interaction and perpetrator connection variables (i.e., amount of interaction regarding the post, amount of interactions that were positive and negative, whether others shared stories with them, similarity between the current workplace and where the SH experience occurred, and whether the perpetrator currently shares a workplace with them); and (3) demographics.

In predicting job satisfaction (Table 4), the models included the 69 participants who posted #MeToo on social media and had complete data. Model 2.1, which included just SH experiences, was statistically significant [*F*_(1,41)_ = 8.49, *p* < 0.01] and explained 17.2% of the variance in job satisfaction. The subsequent models 2.2 and 2.3 were not statistically significant, indicating that none of the context, support, or demographic variables were predictive of job satisfaction. As for life satisfaction as the dependent variable, none of the models were statistically significant (Table 5). In sum, our findings indicate that among people who disclosed #MeToo, SH experiences are predictive of job satisfaction but not life satisfaction, and no additional factors were significant predictors of either.

**Table 4:** Results from hierarchical regression predicting job satisfaction.

**Variable**	**Model 1**		**Model 2**		**Model 3**	
	**B**	**Sig**.	**B**	**Sig**.	**B**	**Sig**
Constant	11.50	^***^	12.05	^***^	14.26	^***^
SEQ	−0.09	^**^	−0.09	^**^	−0.09	^*^
Amt of interaction with others after posting			−0.03		−0.04	
Hearing disclosure from others			−2.05		−2.28	
(*base = yes*)						
Amt of negative interactions			0.00		0.00	
Amt of positive interactions			0.00		0.00	
Incident context			0.50		−0.13	
Current perpetrator context			−0.55		−0.93	
(*base* = *not at current workplace*)						
**Gender**						
Man					−2.07	
(*base = Female*)						
**Race**						
Other					−1.34	
*(base = White/Caucasian)*						
**Education**						
College degree and/or more					−0.46	
*(base = ≤ High School diploma)*						
Income					0.14	
*R* ^2^	0.17		0.27		0.39	
*F* for change in *R*^2^	8.49	^**^	0.74		1.65	

N = 69.

* *p* < 0.05,

** *p* < 0.01,

*** *p* < 0.001.

**Table 5:** Results from hierarchical regression predicting life satisfaction.

**Variable**	**Model 1**		**Model 2**		**Model 3**	
	**B**	**Sig**.	**B**	**Sig**.	**B**	**Sig**
Constant	20.84	^***^	20.41	^***^	21.86	^***^
SEQ	0.08		0.09		0.11	
Amt of interactions with others after posting			−0.01		−0.01	
Hearing disclosure from others			−0.91		−1.76	
(*base = Yes*)						
Amt of negative interactions			−0.04		−0.03	
Amt of positive interactions			0.00		0.00	
Incident context			0.43		0.56	
Current perpetrator context			0.22		−0.01	
(*base* = *Not at current workplace*)						
Gender						
Man					1.56	
(*base = Female)*						
Race						
Other					−1.53	
*(base = White/Caucasian)*						
Education						
College degree and/or more					0.74	
*(base = ≤ High School diploma)*						
Income					−0.52	
*R* ^2^	0.06		0.12		0.17	
*F* for change in *R*^2^	2.81		0.33		0.50	

N = 69.

* *p* < 0.05,

** *p* < 0.01,

*** *p* < 0.001.

### 3.3. Qualitative results

A paradigm model was developed based on our results, representing participants who shared and participants who did not share their SH experience using the MeToo hashtag (Table 6). In mapping our study onto a grounded theory framework, we *a priori* required participants to be employed, so employment is the causal condition. Additionally, we required all participants to have SH experiences, which are also analogous to the causal category in the grounded theory framework. The phenomenon of interest for this study was choosing to post or not to post with #MeToo. Thus, in our paradigm model, we were able to describe the context of the SH events that were shared, contributing reasons for sharing the event, and consequences that resulted from posting using #MeToo.

**Table 6:** Paradigm model and example quotes.

**Paradigm model**	**Codes *(example quote)***
Context	Company culture *(It was sort of the culture there and I didn't feel I could go against it then. I also don't share a lot of that stuff publicly, even if others are. I'll promote their voices, but I don't always feel like I need to add mine.)* Societal norms *(I really just didn't think my story would help because it happens all the time too gay people at work)*
Contributing reasons for sharing	Responsibility to report *(We are in a moment of time where women are being listened to about this. Men are being made to listen to the realities that women face on a daily basis. It is important for out stories to be heard.)* Need for support *(I wanted to unite with others who had experiences as well. We need to support one another)* Positive affect *(Because it feels good to get it off my shoulders when I haven't gotten the chance to share it.)*
Contributing reasons for NOT sharing	Temporal distance *(I guess like a lot of women, I just kept it inside of me all these years. It happened so long ago it almost felt like it was “too late” to say anything.)* Didn't fit in with the movement—male stigma/LGBT concerns/not serious event *(I chose not to say anything because I am male and the harasser was female and I felt that the #MeToo movement was bringing important attention to male-dominated abuse and did not want to take attention from it.)* Uncomfortable sharing *(I'm not sure. I think because some of my social media friends aren't really “friends,” they're just “person I use to go to school with,” “friend of a friend I met one time while drunk,” and so on, I don't feel comfortable really posting anything personal about myself.)* Privacy concerns *(I did not share it on a public platform, but would be open to talking about it in a private setting.)* Negative affect about the event *(It's something I try not to think about, because I still feel very ashamed for not standing up for myself.)* Fear of consequences *(I don't want future colleagues knowing about what happened. I am moving into a leadership role and this incident could slow my career growth.)*
Consequences of sharing	Changes in working conditions *(It definitely makes the workplace better as people could get awareness and would [be] afraid to perform any unusual behaviors.)* Little to no change *(My workplace experience stayed about the same. But more people were aware of the situation.)* Affective changes *(It made me feel just a bit more positive)* Social changes *(Posting about my experience drew me closer to the coworkers who responded to it, and I now consider them close friends outside of the office, which made going to work a much more relaxing experience for me.)*

#### 3.3.1. Context

Context refers to properties that are related to the phenomenon (58). Company culture and societal norms were identified as significant properties related to their experience of workplace sexual harassment and decisions to disclose or not disclose using #MeToo (Table 6).

#### 3.3.2. Contributing reasons

For those who chose not to disclose their SH experiences with #MeToo, six contributing reasons for not posting emerged from the responses. These contributing reasons map onto “intervening conditions” in a grounded theory framework. First was temporal distance from the SH event; this arose from people who indicated that the event was long ago and therefore they did not feel compelled to disclose it on social media. A second intervening condition was feelings of not “fitting in” with the #MeToo movement; people reporting this included comments about feeling as though men or LGBT individuals were excluded from participating in the movement and/or that their incident was not serious enough to feel included in the movement. A third intervening condition was privacy concerns, which were exemplified by participants reporting that they did not want others to know about their experience, they did not want to talk about their experience, they do not use social media for that purpose, or other personal privacy concerns. Fourth, negative affect about the event as an intervening condition was shown by statements of feeling embarrassed, shame, and not wanting to think about the event. A fifth theme focused on being uncomfortable sharing, which included negative feelings about posting #MeToo. Lastly, fear of consequences was the sixth intervening condition that covered a broad range of responses. Statements comprising this intervening condition mentioned fear of organizational responses, fear of others' judgments, not being believed or supported, fear of consequences for the perpetrator, negative interpersonal outcomes, and fear of other consequences.

For those who chose to disclose their SH experiences with #MeToo, three intervening conditions emerged from the responses. These included a responsibility to report, need for support, and positive affect. Statements contributing to the responsibility to report code included references to an obligation to share, protecting other women, perpetrator punishment, desire to help others by sharing, and to raise public awareness as reasons for reporting using #MeToo. The need for support code included responses showcasing a desire to gain social support through posting with the hashtag. Lastly, several responses mentioned positive and cathartic emotional expressions as an influence on sharing, and these codes are accumulated under the category positive affect.

#### 3.3.3. Consequences

We only asked those who reported posting #MeToo about the consequences of their disclosure. The consequences that emerged from participants that reported sharing with #MeToo fit into four categories, including changes in working conditions, affective changes, social changes, and little to no changes. Those who reported changes in working conditions mentioned either worsened or improved conditions. Affective changes were also exemplified by both positive and negative changes, such as embarrassment or feeling better after sharing. Social changes were reported in statements of social support, survivor solidarity, and raising awareness. Lastly, some participants reported that there were little to no changes as a result of sharing with the hashtag.

## 4. Discussion

The purpose of this study was 3-fold. First, the study sheds light on factors associated with reporting #MeToo on social media. Second, this study examined how posting #MeToo influenced job satisfaction and life satisfaction. Finally, the qualitative analyses uncovered the decisions that people made regarding #MeToo disclosure and how—for those who did post—these posts influenced them. Together, this mixed methods study provides insights into the decisions that people made in disclosing #MeToo, the factors that influenced these disclosures, and what happened to them if they did so. In this Discussion, we compare our findings to the broader literature, as well as highlight what new contributions our work makes.

### 4.1. Comparison to formal reporting

One of the more interesting findings of this research is the similarities and differences between the informal disclosure of #MeToo on social media and the formal reporting process. Like the research on formal reporting (7, 74), these social media disclosures were influenced considerably by the person's sexual harassment history. This is not surprising, as greater history means that a person should have more to say. Further, people are more likely to disclose with #MeToo when the perpetrator has greater organizational power is consistent with research on formal reporting (7). This is critical because organizations are more likely to minimize the situation and more likely to retaliate against the reporter when perpetrators have higher organizational power (7). That perpetrator's power matters for both formal and informal reporting suggests that power is a critical factor in the target's understanding of the SH situation and its threat to them, as well as a need for some alleviation. Although beyond the scope of this paper, it is worthwhile to consider in future research whether people who posted #MeToo also formally reported their experiences to their organizations and how these reporting processes turned out. It is possible that people turned to #MeToo in part because their prior experiences with reporting to the organization were dissatisfying at best and destructive at worst.

The literature indicates a low rate of formal complaints filed by people who experience SH, with estimates usually below 1% (62, 63), although some studies report higher rates (74). Freedman-Weiss et al. (63) found that the two most common reasons why surgical trainees did not formally report sexual harassment experiences were perceptions that (a) the SH was harmless and (b) reporting would be a waste of time; Kirkner et al. (75) found similar reasons, as well as being unsure if they would be taken seriously, among faculty and staff in higher education. Vijayasiri (74) indicated that trust in the reporting processes in an organization was a key predictor of reporting; note however that this study used cross-sectional data and, as the author acknowledges, trust in reporting is a result of reporting and good organizational responses, as well as a predictor of reporting. Concerns about retaliation, not being taken seriously, and other silencing techniques have been found to suppress formal reporting of sexual harassment (7, 8, 76–78). To some extent, we found similar effects herein. Those who did not post with #MeToo reported concerns similar to those that often result from formal reporting of SH, suggesting that for some individuals reporting *via* #MeToo is akin to formal reporting in the ways that an organization may respond. More specifically, participants in our study largely reported a fear of organizational consequences, such as negative reactions from supervisors, being perceived as a difficult employee, and a potentially slowed career growth, as motivating them against #MeToo disclosure. The results of the qualitative work herein also demonstrated that whereas sometimes little happened following an informal report, negative consequences could also occur; this is again similar to the scant research on formal reporting (7).

However, our qualitative findings also deviated from research on formal reporting. For example, #MeToo disclosers indicated that their workplace improved after their disclosure and that they had stronger relationships with coworkers. This is the opposite of formal reporting research, which showed that reporting sexual harassment is negatively related to supervisor satisfaction (for women and men targets), coworker satisfaction (for women and men targets), work satisfaction (for women targets), and psychological wellbeing (for women targets) (7). Importantly, the negative effects of reporting SH was largely due to the organization's responses to the report (7); reporting informally *via* an online channel, such as with #MeToo, may not hold the same expectations for organizational consequences and therefore relationships between reporting online and job satisfaction may differ from these relationships with formal reporting. On the other hand, the choice to post online vs. (or in addition to) reporting through formal means may be emblematic of a target's concern over the formal reporting process and perceived outcomes, which are clearly important (7, 8, 63, 74, 77). Reporting with #MeToo may still increase retaliation, minimization of complaints, and sexual harassment tolerant organizational climates, further increasing negative job and life satisfaction.

### 4.2. Which characteristics matter in #MeToo: Individual, relational, and/or organizational?

One of the most robust findings in the SH literature is that organizational climate matters immensely to the prevention or promotion of harassment behaviors and this climate again matters when people are considering formal reporting (14, 17, 74, 79). Additionally, relational contexts matter, such as the ratio of men to women in a workplace (17, 80). Less important are demographic characteristics, such as race or gender; once SH history is accounted for, apparent differences in outcomes of SH across demographic groups disappear (39, 56), although there are clear differences in exposure to SH across demographic groups, including sex, sexual orientation, race, age, shiftwork, among others (43, 81–83).

Similar to these findings, our research demonstrated that demographic characteristics had little effect on #MeToo reporting, once sexual harassment history was accounted for. They also had little effect on job satisfaction or life satisfaction following #MeToo disclosure. Finally, in our qualitative analyses, we did not detect an effect of demographics in the responses, other than the theme “not fitting in to the #MeToo movement.” However, this latter finding has some parallels to the SH literature that shows that exposure to SH differs across various demographic groups but the effects of said exposure does not (i.e., outcomes are due to differential exposure rather than differential vulnerability), such as #MeToo movement can be perceived as a way for women to highlight male-perpetrated harm and that SH often takes the form of male perpetrators harassing women targets (17). However, it is clear that SH does not just happen to women and is not only perpetrated by men (13), so while #MeToo might particularly focus on this situation, it is important to create spaces for informal disclosures of SH with other configurations as well as ensure that formal reporting processes do not make assumptions about perpetrator and victim characteristics.

Relational factors appeared in our research, as they have in other SH research. However, our mixed methods research was able to expand our understanding of the relationships that matter to people when they are thinking about disclosures. Not only does the power of the perpetrator matter (7, 38), but also the extent to which relationships with the perpetrator, other coworkers, and the organization are on-going. Notably, the informal disclosures studied herein might be unusual in that the similarity with the workplace when the harassment occurred makes #MeToo disclosures more likely, but it is as yet unknown whether this is also the case for formal reporting. Further, our results show the relational effects of disclosing *via* #MeToo and how this prompted better relationships with coworkers, such as expressions of support. Our respondents also indicated improved working conditions, such as greater awareness of the problem of SH. These responses to the SH by coworkers may be important to future prevention and current responsiveness, and while they cannot make a person “whole” following SH (84), this support might alleviate some of the negative outcomes that the target experienced.

However, our results also demonstrated that there were some negative effects of disclosure, such as worsened working conditions. Additionally, one of the major themes in why people felt discouraged from reporting was fear of consequences such as retaliation or being labeled a “troublemaker.” These findings are consistent with research indicating the reasons that people do not formally report SH (63, 74, 77). Ultimately, as one of the major themes of our qualitative work shows, organizational culture is a key contextual factor that frames people's understanding of SH and SH disclosures.

### 4.3. More than a bandwagon: Complex decisions for disclosure and non-disclosure

Overall, the quantitative portion of our study suggests that experiences of sexual harassment–rather than social media use–were the strongest predictor of #MeToo disclosure, indicating that #MeToo disclosures were not merely some social media bandwagon that people hopped on, but a reflection of events that accumulated across a person's lifetime. Consistent with this finding, geospatial analysis of the use of #MeToo on Twitter, showed that certain geographic regions received higher rates of disclosure, suggesting that those places in greater need of violence-related resources were likely to have more posts (85). Our finding is also in line with previous research on disclosure of distressing experiences on social media, which indicates that these sorts of posts may be deemed inappropriate by others (86, 87), and therefore less likely to receive public disclosures at all. Supported by our qualitative findings, participants may have decided to disclose these distressing experiences because they anticipated supportive responses and reactions from others in their network (86, 88). Further, the idea that #MeToo disclosures were made by those who simply hopped on a social media bandwagon is a reflection of the stigmatization seen in other instances of social media disclosure, the effects of which lead individuals to non-disclosure. Previous research on stigmatized identities, in particular those pertaining to mental health, have shown that there is stigma associated with social media disclosures, and that individuals may choose non-disclosure in order to avoid being labeled as attention-seeking (89). Whereas, the social media context has been consistently shown to limit distressing disclosures for reasons related to stigmatization and privacy (86, 89), it is aparent in the current study that sexual harassment experiences drove decisions to disclose using #MeToo.

Moreover, continued connection with the perpetrator resulted in greater likelihood to disclose experiences with sexual harassment. This is likely a reflection of the amplification of distress experienced by those who must still remain in contact with their perpetrator, whether this indicates ongoing harassment or a permissive culture around sexual harassment in one's organization. Further, our results indicate that power dynamics, but not demographic characteristics, influence #MeToo reports, such that targets whose perpetrator has more organizational power are more likely than targets whose perpetrator has equal organizational power to create a #MeToo post on social media. These results may be indicative of individuals sharing their most negative experience with sexual harassment using the hashtag. Previous research has shown that sexual harassment from perpetrators with greater organizational power is perceived as more negative and stressful (90, 91), and therefore may be the incident that individuals are most likely to share. Further, the nature of the #MeToo movement was exemplified by targets with lower power (e.g., Alyssa Milano) than their perpetrators (e.g., Harvey Weinsten) and posts may have been more likely to follow that template.

Our qualitative results further developed our understanding of the factors that influenced decisions to post #MeToo. Some of these results aligned with the quantitative results, including having a company culture that was tolerant of frequent sexual harassment. Our qualitative results also expanded our understanding of these influences by illuminating factors that were not asked on the quantitative portion of the survey. In particular, participants who disclosed using #MeToo frequently reported a sense of moral responsibility in supporting the #MeToo movement as a motivating factor in their decision to post. This finding may be indicative of individuals viewing the #MeToo movement as online activism, often described as “hashtag activism” whereby individuals engage in conversations on social media pertaining to pressing social issues (92, 93). Further, previous research suggests that targets of sexual victimization may disclose on social media to raise awareness about gender-based violence, as well as prevent future abuse (94). Additionally, #MeToo posters were driven by a desire to receive support from others with similar experiences, and believed that the cathartic nature of posting would ultimately “feel good.” Previous research supports this finding, indicating that hashtag use can allow individuals to build supportive networks (95, 96), while specific disclosure of sexual assault on social media is often motivated by failed support from other individuals in one's life (25). On the other hand, those who did not disclose using #MeToo cited the opposite; that posting would ultimately “feel bad” and trigger shame or embarrassment over the event. For some of these participants, the event was perceived as happening too far in the past, or too near in the present to comfortably allow for sharing. This finding is in line with the current understanding of social media non-disclosure of stigmatized identities and distressing experiences in contexts outside of sexual harassment (86, 87, 89). Further, participants who were not #MeToo posters largely reported feelings of discomfort around sharing SH experiences online, with many participants directly referencing privacy concerns as a motivating factor in their decisions not to post. Another major concern that halted disclosure was the fear of organizational and social consequences or repercussions that could arise from sharing a #MeToo post. Taken together, these motivations toward non-disclosure using the hashtag are consistent with previous research on strategies used to manage risk when using social media, such as self-censorship, not participating in social media at all, and content regulation (86, 97–99).

### 4.4. The power of exploratory, mixed-methods research

In our quantitative survey results (Research Question 2), we found that SH history affected job satisfaction but not life satisfaction. This is likely because overall life satisfaction is determined by many factors outside of the work context. We also found that no other quantitative variables that we measured predicted job or life satisfaction among those who disclosed #MeToo. In contrast, our qualitative findings demonstrate that the consequences of sharing affected both job and life domains. #MeToo posters reported the consequences of sharing their SH experience ranged from little change at all to changes in working conditions, social support, and affect. In the job domain, some #MeToo posters reported a perception that supervisors and coworkers became more aware of the pervasiveness of SH and that working conditions improved as a result of this awareness. Further, #MeToo posters specifically reported positive social changes as a result of their posts, such that social support increased. In the life domain, #MeToo posters reported that they felt a more positive affect as a result of posting.

Together, these results point to a broad model of the phenomenon of #MeToo disclosure. As noted and as required by our study, a #MeToo posting requires sexual harassment experiences. Posting #MeToo has a number of factors that contribute to it, including individual factors (e.g., SH history, gender), social media factors, interpersonal relationship factors, and organizational factors. In drawing upon both the quantitative and qualitative findings to build our model, the most important factors leading to #MeToo disclosure were pervasiveness of sexual harassment experiences, both personally and as a result of company culture, a continued connection with the perpetrator, and having a perpetrator with greater organizational power. Further, perceptions of a moral obligation to report, that responses to posting would be supportive, and that affect would improve after posting were motivating factors toward reporting. The main motivating factors against reporting were negative affective feelings about the SH event specifically, and negative feelings around reporting the event in a public forum. Additionally, a fear of organizational and social consequences hindered #MeToo posts. In summarizing the outcomes of #MeToo posting from our mixed-method design, the outcomes of posting with #MeToo are largely influential to job satisfaction, with changes to working conditions and social support changes indicated as the most likely affected factors.

### 4.5. Practical recommendations

Ultimately, our results reify what has been long stated by SH researchers and anti-harassment scholars: SH prevention is needed, and swift and effective resolutions to SH when it occurs is necessary. Our results demonstrate that organizations should concentrate on preventing SH given that this was the primary (negative) predictor of job satisfaction. Further, experiencing positive support after a #MeToo posting showed no ameliorative influence, indicating that prevention is critical because good support does not seem to be sufficient to make people whole after the injury of these events (7, 84, 100).

However, we must also caution against workplaces monitoring social media for informal reports of sexual harassment. It is important that harassment targets have agency in their responses to SH, and it is important that workplaces allow workers to have privacy. Unfortunately, some people end up in a difficult place—they know their coworkers have had some SH experience, and they want to respect their choice not to report, but they might have rules at work about mandated reporting. We believe that policy is needed in this arena and that this policy is clearly articulated to workers. At this moment, case law in the United States, for example, indicates that when organizations know or should have known about sexual harassment and do not act, then they are potentially liable (Burlington v. Ellerth; Faragher v. the City of Boca Raton); thus, when employees–especially supervisors–become aware of sexual harassment claims, even though social media, it is possible that the organization will be seen as “knowing.” In such instances, it is probably sensible for the relevant HR function to be notified and for them to approach the person who made their social media post about their harassment experiences. That person could then decide to pursue a claim or not, and HR could investigate based on their information or close the case. This should meet the employer's obligations, as this would demonstrate the organization's attempts to alleviate the SH.

### 4.6. Limitations

Some limitations should be acknowledged. The content of the #MeToo posts were not examined. This is vital as the content of #MeToo posts may provide further information regarding the impact of harm from the reported event. Furthermore, the act of writing a #MeToo post may provide some ameliorative effect on psychological outcomes and influence one's job or life satisfaction. For instance, posting more information on social media may give those who post the opportunity to process their feelings on the damaging events. Specifically, research has shown that expressive writing can provide benefits to reduce psychological distress (101).

Additionally, given the low sample size across demographic groups, the current study was unable to investigate differences among races, ethnicities or sexual orientations; relatedly, we could not discern how multiple minoritized identities intersected and affected people's experiences. Finally, the time that elapsed between #MeToo in September 2017 and our data collection in June 2018 means that intervening events—both more harassment as well as addressing and ameliorating events—could have dampened or heightened relationships between #MeToo and outcomes.

### 4.7. Suggestions for future studies

Future research might explore the experiences of reading #MeToo or similar posts from personal connections, such as friends and coworkers, and how this influences wellbeing. Additionally, given the increasing role that social media plays for businesses today as well as the increasing monitoring of employees' social media presence, a necessary future study would investigate how monitoring impacts the reporting of sexual harassment. This particular line of research may be of interest as the posting of reports online may impact hireability, promotability, and other outcomes for those who share online. Finally, sexual harassment is not confined to the United States and #MeToo is a movement that occurred beyond its borders. For example, France had a similar social media campaign, #BalanceTonPorc [#exposeyourpig; (102)]; China, on the other hand, has seen SH targets sued by the people they accused; there are also concerns about the safety and freedom of people who publicly accuse powerful Chinese official of sexual misconduct (103). There are also industry- and community-specific #MeToo movements, such as #MeTooPhD (104) and #ChurchToo (105). Further research into these linked movements is necessary, such as how posting #MeToo (or similar) affects people. Future studies that are needed would explore a variety of factors, such as sociosexual power hierarchies, women's rights within a country, and unfettered access to the internet, might be especially important to research across cultures. Across industries and communities, similar factors could include sociosexual power hierarchies, leadership, policies against sexual harassment, and external accountability bodies.

The findings on social media privacy indicate a direction for important future research as physical privacy allows for more likelihood of reporting for women (106) but those with public social media profiles were more likely to post online. As such, one study that is needed would focus on how considerations for privacy are addressed by investigating physical privacy vs. online privacy in regards to reporting. This, too, is likely to vary across cultures, ages (e.g., teens are likely to be monitored by parents), industries, and the like.

## 5. Conclusion

This study examined the informal reporting of sexual harassment through social media. The findings suggest that disclosure *via* social media is similar to formal methods of reporting. Namely, disclosure *via* social media is largely predicted by SH experiences, current connection with the perpetrator, and relative power dynamics. In addition, disclosure of SH *via* social media was predictive of job satisfaction, but not life satisfaction. Social media has changed the way people communicate with each other and creates new ways by which people can interact and disclose information. Researchers should continue investigating informal reporting across platforms to determine how informal reporting impacts targets and organizations. Most importantly, sexual harassment experiences were the strongest predictor of #MeToo disclosure. This finding should push organizations to recognize the legitimacy of social media reports of sexual harassment. Instead of minimizing or retaliating against those who disclose sexual harassment through social media, organization leaders should recognize that these reports come from those who are most affected by this harassment. They should also consider the possible failings of their formal reporting systems, their impact on the target's job and life satisfaction, and the reasons why employees choose to disclose online.

## Data availability statement

The raw data supporting the conclusions of this article will be made available by the authors, without undue reservation.

## Ethics statement

The studies involving human participants were reviewed and approved by Texas A&M University IRB. The patients/participants provided their written informed consent to participate in this study.

## Author contributions

RS performed the statistical analysis. All authors contributed to conception and design of the study, wrote sections of the manuscript, and contributed to manuscript revision.

## Footnotes

^1^By social media, we mean Web 2.0 applications, like Twitter, Facebook, and Instagram, which allow users to create and share content with other users. Note that social media can be anonymous or connected with a person's real identity. Additionally, some forms of social media allow a person to limit who can see their posts (e.g., privacy settings on facebook) whereas others are more open and available to people (e.g., posting on a Reddit forum).

^2^Five non-binary participants were removed from the analysis due to low sample size, however the logistic regression including these participants revealed that non-binary individuals were 9.16 times more likely to post with #MeToo than were men (*p* < 0.05).
